# The Importance of Accurate Host Species Identification in the Framework of Rabies Surveillance, Control and Elimination

**DOI:** 10.3390/v14030492

**Published:** 2022-02-28

**Authors:** Paola De Benedictis, Stefania Leopardi, Wanda Markotter, Andres Velasco-Villa

**Affiliations:** 1 FAO Reference Center for Rabies, Istituto Zooprofilattico Sperimentale delle Venezie, 35020 Legnaro, Italy; sleopardi@izsvenezie.it; 2 Centre for Viral Zoonoses, Department of Medical Virology, Faculty of Health Sciences, University of Pretoria, Pretoria 0001, South Africa; wanda.markotter@up.ac.za; 3 Centers for Diseases Control and Prevention, 1600 Clifton Rd. NE, Atlanta, GA 30333, USA; dly3@cdc.gov

**Keywords:** wildlife reservoir, host identification, epidemiology, rabies, infectious diseases

## Abstract

Accurate host identification is paramount to understand disease epidemiology and to apply appropriate control measures. This is especially important for multi-host pathogens such as the rabies virus, a major and almost invariably fatal zoonosis that has mobilized unanimous engagement at an international level towards the final goal of zero human deaths due to canine rabies. Currently, diagnostic laboratories implement a standardized identification using taxonomic keys. However, this method is challenged by high and undiscovered biodiversity, decomposition of carcasses and subjective misevaluation, as has been attested to by findings from a cohort of 242 archived specimens collected across Sub-Saharan Africa and submitted for rabies diagnosis. We applied two simple and cheap methods targeting the Cytochrome b and Cytochrome c oxidase subunit I to confirm the initial classification. We therefore suggest prioritizing a standardized protocol that includes, as a first step, the implementation of taxonomic keys at a family or subfamily level, followed by the molecular characterization of the host species.

## 1. Patching the Way for Appropriate Epidemiology of Multi-Host Pathogens

Appropriate identification of the animal host(s) that play a central role in maintaining circulation of human or animal pathogens is essential for the control, prevention and elimination efforts, as well as to unveil critical details on the ecology of zoonotic infectious diseases and conservation strategies. We refer particularly to pathogens with a wide range of susceptible hosts that cannot only be transmitted between multiple host species, but also have the inherent ability to get established in new susceptible host populations when they are naturally (climate change, natural disasters) or artificially (human-mediated) introduced to novel ecological niches, thus creating a new reservoir. Among them, zoonotic pathogens, that are capable of infecting humans, have received attention increasingly over the last decades. SARS-CoV-2, the most recent pathogen responsible for a devastating COVID-19 pandemic and Ebola, with recurrent outbreaks that have spread out of Africa, constitute remarkable examples [1,2]. However, although efforts have been devoted to rapidly and fully characterizing emerging pathogens in humans, the complex ecological mechanisms leading to the establishment and maintenance of a pathogen in the animal kingdom are being unveiled with difficulty. Current evidence indicates that most emerging diseases in humans are of zoonotic origin and are mostly the result of a direct or indirect spillover from wildlife [3,4].

Conversely, our understanding of multi-host pathogens, and their origins in wildlife, is often poor because of our lack of attention to critical processes such as infection susceptibility, host range, temporal dynamics, clinical progression, morbidity, mortality and pathogen evolution [5]. Failure to establish a comprehensive understanding of a pathogen at its source can hamper policy formulation and lead to ineffective or counterproductive control measures with costly implications for public health as well as wildlife conservation [6,7].

Among multi-host zoonotic viruses, those pertaining to the *Lyssavirus* genus, causing an acute clinical encephalomyelitis in mammals, affect a remarkable number of natural hosts in wildlife populations [8]. The rabies virus (RABV), the genus’ prototype species that is responsible for most of the human and animal rabies cases worldwide, has established independent transmission cycles in certain mammal species primarily through host shifts, thus overcoming host species, geographic and ecological barriers [9,10]. Currently, the control of canine rabies represents the main objective of national stakeholders and international organizations that have been all collaborating towards a final goal of zero human deaths due to dog-mantained rabies by 2030 [11]. The optimization of the few resources available to achieve such a goal implies that in dog-rabies endemic areas the surveillance of wildlife is mostly limited to cases of human exposure while the spatial and temporal dynamics of rabies infection in these hosts is poorly investigated. However, there is a growing interest to discriminate whether wildlife, especially wild canids, can maintain independent rabies’ cycles (of dog or bat origin) in parallel within or around dog-rabies endemic areas, or, conversely, if they are accidental hosts with scarce epidemiological importance [12].

RABV circulation in wildlife not only poses a significant threat for conservation of endangered mammals but also a latent risk for the re-establishment of rabies’ cycles in unvaccinated domestic animals, which renders rabies as an authentic priority health concern [13,14,15,16]. Based on a literature review, rabies is reported in about 200 mammals, and of those, sixteen are classified as endangered [17]. Rabies also represents a direct conservation threat for two African canids, namely the Ethiopian wolf, *Canis simensis*, and the African wild dog, *Lycaon pictus*, which have been affected by the spillover of endemic rabies in domestic dogs [17].

Despite the importance of appropriate host species’ identification, very few lyssavirus surveillance efforts have coupled virological and host identification approaches, thereby jeopardizing the early detection and accurate identification of putative new host species for both RABV and non-RABV lyssaviruses [18,19,20,21,22,23]. 

Rabies’ diagnostic laboratories usually implement standardized identification using taxonomic keys (classic taxonomic methods). However, in certain rabies endemic areas with enormous biodiversity [24], there is an implicit increased risk of misidentification of the reservoir hosts. For example, a novel rabies variant circulating in wildlife in Mato Groso, Brazil, was retrospectively described in a wild kinkajou (*Potos flavus*) that was initially misarchived as a bat and further recalled by the diagnostic laboratory as a monkey [18]. Although specimens of such a case were the individual were identified through partial sequencing of both the Cytochrome b (Cytb) and the cytochrome c oxidase subunit I (COI) partial sequencing [25,26], no rabid kinkajous had been recorded in Brazil thus far, meaning that this individual was likely mis-identified from submission. Interestingly, at the same location in the same period, a specimen referred to as a capuchin monkey (*Cebus apella*)—with no further genetic confirmation or information as to how the animal was identified to species—harbored the same novel rabies variant as the kinkajou [18,27], raising doubts on whether they were two different specimens or rather a unique finding tested and characterized by two independent groups. 

Similarly, in large ecological studies samples can be misidentified or mislabeled leading to the exportation of tissues potentially containing select agents or endangered species that may require special permits. Whatever the case, these involuntary mistakes may compromise large investigations leading to the destruction and elimination of tissues compromising the robustness of large scale eco-epidemiological studies [23]. Thus, herein we urge the prioritization of the implementation of classic taxonomic identification tools, by trained and experienced personnel, to identify suspected rabid animals to family level whenever possible. This will subsequently permit us to go deeper into the identification at a species level through molecular methods. To support with data our point, we randomly chose samples from a large repository to assess how frequent specimens had been species misidentified and compared this information with molecular identification methods. In addition, we describe the application of two feasible molecular alternatives to identify host species, from brain tissue in carcasses submitted to a rabies’ diagnostic laboratory as well as expose some of their limitations in countries where the extant of the mammal biodiversity has been poorly described.

## 2. Molecular Methods for Hots Species Identification 

We randomly selected 242 brain specimens collected from ten countries in Sub-Saharan Africa, among which 127 were Rabies positive (52.5%). Selected specimens were actually part of a wider repository of samples at IZSVe, which were voluntarily shipped to the IZSVe from veterinary laboratories across Africa requesting services we provide to rule out rabies diagnosis and genetic typing of rabies virus and other lyssaviruses in our capacity of the International Reference Collaborative Centre for rabies diagnostics and research. Most collaborating laboratories have implemented their own animal identification protocols for all samples (carcasses) received before running rabies diagnosis. Although not requested by our customers at the time of submission, we included molecular identification of host species in most samples received for rabies diagnosis and genetic typing. It is noteworthy that most specimens received at IZSVe are not part of systematic surveillance activities where robust samplings had been conducted to answer specific or broad questions on rabies virus and its host diversity, rabies epizootiology or its ecology. Rather, these samples mostly came from random samplings from potential cases involving human exposures. For the scope of this perspective, information on the country of origin for each of the samples is not relevant.

We recommend molecular identification of mammal species to complement classical taxonomic identification approaches as follows. The extraction of RNA should be conducted from brain tissue using the NucleoSpin RNA kit (Macherey-Nagel, Düren, Germany). This procedure allows the concomitant detection of lyssavirus nucleic acid for rabies diagnosis and the molecular identification of the mammal host species. PCR reactions are carried out using the OneStep RT-PCR kit (Qiagen, Hilden, Germany), RNase Inhibitor (Promega, Madison, USA) at final concentration 0.4 U/µL and 0.4 µM of each primer COIBATF1 (5′-TCAACYAATCAYAAAGATATYGGCAC) and COIBATR1 (5′- ACTTCYGGGTGRCCRAARAATCA) [28]. The thermal cycler program was 30 min retro-transcription at 50 °C, 1 cycle of 15 min at 95 °C, 45 cycles of 30 s at 94 °C, plus 30 s at 52 °C, plus 40 s at 72 °C and final elongation of 5 min at 72 °C. Amplicons were sequenced using Sanger’s approach and we relied on the BOLD system as the reference database for the host identification [29]. In cases where the COI sequence did not show sufficient identity for BOLD identification, we used the BLAST online platform (Rockville Pike, USA) to identify the mammalian species with the highest genetic identity. When the amplification or sequencing for COI failed or when the identification using the COI gene was inconclusive, we attempted amplification and sequencing of a second locus, of the cytochrome b (CytB), following the same molecular approach and using primers available from literature [26]. As CytB is not included in the BOLD system, we always relied on BLAST analyses for this locus, considering a reliable identification genetic identity higher than 99%.

## 3. A Pragmatic Example of Rabies Surveillance Coupled to Host Identification in Terrestrial Carnivores in Africa

In the present study we included *n* = 242 samples from 14 different hosts, as identified by the submitting laboratory. Among these, *n* = 203 were defined at species level (ten species) whilst *n* = 39 (16.1%) were defined as subfamily only (namely, bovines, caprine, canines and felines) or with common names that were associated with more than one wild species (namely, jackal and wildcat) (Table 1). Among these samples, the species assigned after the genetic characterization matched with the initial subfamily definition and all jackals turned out to be highly related with the side-striped jackal (*Canis adustus*). On the other hand, a sample labelled as a wildcat was identified as a common genet (*Genetta genetta*) with 100% identity on COI gene. Overall, we detected host misidentification in 9.9% (24/242) of the sampled individuals across five countries (Table 1). 

Overall, the great majority of samples 82.2% (199/242) were referred to hosts in the family Canidae, with domestic dogs comprising 80.6% (195/242). This result is consistent with the fact that the dog is still the most important reservoir for rabies in this region of the world. Samples presumably obtained from dogs were misidentified in 8% (16/195) of the cases. Strikingly, the genetic analyses confirmed their association with six different species, including an African golden wolf (*C. anthus*), five domestic cats, a donkey, a cow and a goat and a patas monkey (*Erythrocebus patas*) (Table 1). Conversely, morphological identification of wild canids was mostly correct, even if samples were generically attributed to “jackals”. We only found two exceptions, one sample referred to as “fox” that was genetically compatible with a side-striped jackal, with the highest identity shown with *C. adustus*, and one African golden wolf that was misidentified as a domestic dog (Table 1). Based on the genetic identification results, the percentage of canids sent to the laboratory for rabies diagnosis was slightly lower than originally declared, accounting for 76.8% (186/242), with 74.8% (181/242) confirmed as domestic dogs. 

Hosts referred to species level within the subfamily Felinae only comprised 2.9% (7/242), which included five domestic cats, a wildcat and a lion. Among these we noticed only one misidentification, where a common genet (*Genetta genetta*), belonging to the family Viverridae, was labelled as a wildcat (Table 1). However, it is crucial to note that all samples showed identity values higher than 99% with the domestic cat (*Felis catus*), the African wildcat (*F. silvestris lybica*) and the sand cat (*F. margarita*), which confirms that all were misidentified as European wildcat (*F. silvestris*). Given that, the use of a single locus such as COI is not accurate enough to provide a robust taxonomic classification of the genus *Felis,* two samples referred to as felids were further characterized using the CytB. Results, however, showed ambiguous lower identities between 96 and 97% with *F. catus, F. silvestris lybica* and *F. silvestris* and of 95% with *F. margarita* that did not allow an accurate host identification. The relatively frequent occurrence of feline hybridization between the African wildcat and a broad spectrum of domestic cat breeds is a well-recognized phenomenon, that has given rise to several unidentified cat hybrids in South Africa. This hybridization process has been linked to uncontrolled human population growth that has resulted in severe fragmentations of natural habitats with the subsequent introduction of domesticated animal species [30]. We suggest that this phenomenon has dual implications, not only for public health, but also for the conservation of threatened species. Moreover, five cats were labelled as dog samples, thereby increasing the real percentage of felid samples from 2.9% to 4.1%.

Overall, 14.5% of the samples (35/242) were originally identified as livestock, which encompassed 9% of cows (22/242), 5% of goats (12/242) and a donkey. Unfortunately, these samples presented the highest number of mismatches between the declared and genetically-confirmed host species. Among the 35 declared livestock samples, 14.3% (5/35) were identified as a different species, these comprised two dogs that were originally recorded as a cow and a donkey and three goats genetically identified as cows (Table 1). In addition, ten samples collected from cattle were incorrectly archived/mislabeled as dogs (*n* = 7). The final proportion of livestock samples was 17.4% (42 samples out of 242) instead of 14.5% as declared.

Misidentification of specimens in the sample dataset tested might have been attributed to two types of errors: (i) specimen or animal misidentification at the time of submission or (ii) incorrect archiving or mislabeling of tissues (i.e., domestic/livestock animals and species belonging to subfamilies roughly distinguishable (i.e., bovines vs. canines)). The latter type of error was particularly evident by the mismatch between two samples from a dog and a donkey archived by a single laboratory. As for the incorrect identification of carcasses, this was most common among canids and felids that might have occurred at the step of brain removal or during the collection of brain specimens. Brain removal is often conducted in the field to reduce the final volume during transportation and for optimization of storage space. Animal carcasses, especially those collected from wild animals during passive surveillance activities (road kills, remains from depredated animals, dead animals partially eaten by scavengers in which proper observation of suspect rabies signs was not possible) are often highly decomposed, severely mutilated or mummified, hampering their correct identification by field staff. Furthermore, in most cases the field staff handling carcasses do not have the proper training or the level of expertise to identify animals to species level, in particular those that are not domestic species. Even in the case when the entire carcass is submitted to a veterinary diagnostic facility or laboratory, diagnosticians might not have been appropriately trained to recognize or identify wild animals. We must emphasize that viral isolation in mice or cell culture is still a common and/or necessary practice to isolate and amplify the original virus if the brain material available is scarce. This procedure prevents any further investigation to genetically identify animal species from the primary source of the virus. In any case, given the high and still undiscovered biodiversity of the continent, we suggest that tissue sample archiving might benefit from an initial classification of the carcass at family level, especially for wild animals or decayed carcasses and then a further identification through molecular testing.

## 4. The Drawback of Biodiversity

Overall, appropriate identification of a wild animal is much more prone to mistakes than the identification of domestic animals. This is even more notorious in resource limited areas or regions with high but often poorly characterized biodiversity. Conversely, adequate standard operating procedures on quality control for labeling and recording of specimens associated descriptive/identification data in databases should be in place at the field and in the laboratory setting to prevent labelling and recording mistakes from the beginning.

Host misidentification inevitably leads to the misunderstanding of the complexity of rabies epidemiology/epizootiology in certain areas, which is paramount for the early detection of emergent rabies’ cycles often associated with an unexpected or newly recognized reservoir-host species. It is also important to keep in mind that the incorrect identification of a reservoir-host species and its legitimate recognition may also have direct consequences for the accurate reconstruction of the spatial-temporal evolutionary stories of RABV variants, lineages or clades that ultimately hamper an appropriate risk assessment to plan the most effective control, prevention and elimination activities in the field.

To contextualize the importance of the misidentification of several wild animals in a small subset of samples tested for this study, we will focus this section of our discussion to a misidentified specimen infected with the Africa 3 RABV clade, which was initially reported as a honey badger (*Mellivora capensis*). However, after genetic characterization, using BLAST, this specimen turned out to be genetically distant (82%) to the existent honey badger sequences available in the GenBank CytB database (no match in the COI database), but slightly similar (84.7%), yet too divergent, to the clawless otter (*Aonix capensis*), depending on the gene investigated (Table 1). The exact species’ identity of this individual host would still be unclear based on partial sequencing that has not been coupled with classical taxonomic identification approaches. Thus, our findings underline the lack of representation, in sequence data bases and in taxonomic catalogs, of the extant biodiversity of honey badgers, as well as many more mammal species and subspecies across Africa, and within similar megadiverse regions worldwide. With limited sequence data available and the poor taxonomic classification of extant species, selecting one species over another, based on low sequence identity, represents a blindfolded process likely resulting in the misidentification of the implicated mammal host species. This widespread practice subsequently affects the understanding of the actual complexity of the rabies virus ecology, epizootiology, which in turn has severe consequences on selecting or designing the most effective control, prevention, conservation and elimination measures. In this particular example, there are several biological differences among the clawless otters and the honey badgers, the latter being often associated with rabies in Southern and Eastern Africa, while the former has been barely reported as rabid globally. Although they are both large mustelids and primarily carnivores, they differ substantially in habitat, natural distribution, diet and overall behavior. There are several recognized subspecies of honey badgers, whose natural range spans across Sub-Saharan Africa, Morocco, the Arabian Peninsula, and the Indian subcontinent. They feed on a wide range of vertebrates and might become notoriously aggressive, especially if attacked. Conversely, the African clawless otter permanently inhabits water bodies in the savannah and lowland forests across Sub-Saharan Africa with a diet mainly comprising water-dwelling animals. In this context, correct attribution of the host is paramount to interpret the epizootiologic and genetic data, including the circulation of the different RABV lineages, variants or clades associated with a complex assemblage of subpopulations within a host species or closely related species, each one characterized by particular geographical, ecological, biological and behavioral features [6,9,10,31,32]. Similarly, a reported wildcat (*Felis lybica*) infected with the same Africa 3 RABV clade was retrospectively identified as a common genet (*Genetta genetta*). Although both the wildcat and the common genet belong to the suborder Feliformia, they are phenotypically and genetically differentiated into two families. Important eco-epizootiological differences among the two host species include the higher opportunity for a wildcat to come into contact with domestic cats, and potentially hybridize [30], a phenomenon mainly enhanced by human encroachment. These two revisited hosts’ identifications underline the complexity and poorly explored epizootiology of the Africa 3 clade RABV. This RABV major clade has been characterized by apparently faster/broader evolutionary rates, compared to the other major RABV clades from the dog-related group [32]. Thus, the strikingly high genetic heterogeneity of the Africa 3 clade, widely discussed in the past, could have been associated to the natural infection of a large variety of wild carnivores in a highly complex hyper-diverse ecosystem rather than to a long evolutionary story [33]. In this context, the incorrect identification of the host species might have paramount implications for the epizootiological forecasting of the complex ecology of this RABV major clade. Indeed, although the yellow mongoose (*Cynictis penicillata*) has been suggested as the natural primary reservoir of Africa 3 RABV (also called “African mongoose RABV variant”), other wildlife species (making a complex assemblage of concurrent reservoir hosts) might be also playing an important role in the perpetuation or maintenance of independent rabies cycles (associated with lineages of the Africa 3 clade), which also has critical implications in determining the extent of the geographic spread of this variant and for the effective design of rabies control, prevention and possible elimination strategies. 

In a larger subset of samples, we noticed a general pattern for identification at the subfamily level, which included the utilization of coarse, often ambiguous terms that mislead the accurate identification of domestic versus wild animals. Outstandingly, we discovered a sample from the African golden wolf among a group of specimens identified as dogs. Similarly, we detected four individuals closely related (but not fully resolved) as side-striped jackals (*C. adustus*) among a group of samples originally identified as fox (*n* = 1) or simply referred as “jackals” (*n* = 3). A similar pattern of misidentification in jackals originally identified as foxes or domestic dogs has been notified in Sub-Saharan Africa [23]. Our findings stress how poorly investigated the animal biodiversity, from the genetic point of view, in the African continent is, as well as the limitations for the correct taxonomic identification of animals in highly biodiverse underserved regions where human encroachment is dramatically increasing, favoring crossbreeding of domestic with wild species and giving rise to previously unseen hybrid species. Thus far, the extant genetic and taxonomic diversity of mammalian species across the African continent is likely under-investigated and subjected to continuous reclassification. This is evident on the recent redefinition (almost exclusively on a genetic basis) of the African golden jackal, which was recently defined as the African golden wolf (*Canis anthus*), which is a completely distinct species from the Eurasian golden jackal (*Canis aureus*) [34]. Notably, Koepfli et al. [34], noticed striking morphologic similarities between the East African and Eurasian golden jackals that might have confounded taxonomists thus far. In fact, Africa accounts for several canid species that might have been only partially resolved both genetically and taxonomically. In this context, it is noteworthy to notice how current databases might still include sequences with outdated classifications, which increases challenges for a correct identification, even using molecular tools. In our case, the identification of *C. anthus* using COI sequence homology was initially challenged by a 100% identity with two sequences uploaded in the BOLD repository as *C. aureus*, whose natural range does not comprise the area where the specimen of interest was found [34]. It is then crucial that scientists refrain from submitting sequences which have not included rigorous supporting evidence of correct taxonomic identification based on strict and robust morphological keys. In addition, researchers should be encouraged to update the taxonomic resolutions according to re-classification of certain taxa. Multidisciplinary investigations involving infectious disease experts, mammologist and taxonomists should be encouraged to warrant a better understanding of the ecology of infectious diseases. Moreover, nucleotide sequence databases and repositories should increase quality control policies to improve the accuracy of molecular identification tools.

Finally, free roaming canine populations are distributed almost invariably across the African continent with a high possibility of contact and even hybridization with wild canids. Thereby, hybridization of the species should be considered when approaching disease control and elimination plans. The complex RABV evolutionary story between dog and wildlife populations has been previously suggested in the U.S.A. [31], China [35], Turkey [36] and more recently in Iran [37] and Brazil [38]. Our data indicate that a similar picture can be drawn for some wild cats and hybrids across Africa as well. On the other hand, a recent modelling study indicated that in South-East Tanzania a third of the inferred rabies transmission events might involve wildlife-to-wildlife transmission, with sustained transmission chains within jackal populations [39], although in this particular case species identification was defined on an interview basis. Similarly, the recurrent identification of a specific rabies sub lineage in Ethiopian side-striped jackals over two years potentially indicates a rabies cycle diverging into wildlife [23]. In this specific case, a genetic characterization of partial Cytb was attempted by Binkley et al. [23], in the absence of a host full resolution. However, we predict that is not possible to determine the role of wild canids without genetically identifying the hosts included in modelling analyses. Thus, adequate training of biologists, mammologists should be part of the standard at infectious diseases laboratories across the world.

## 5. Conclusions

Overall, our findings support longstanding empirical observations on the high frequency of species misidentification in deceased or euthanized animals with suspected rabies. This, along with recent research [18,23], underlines a chronic problem that requires urgent attention to advance understanding of the ecology of RABV transmission, not only within dog populations, but also within sympatric wildlife reservoirs that may play either a central or a supportive role in the maintenance and ecological persistence of the disease. Thus far, most studies performed do not substantially crosscheck the actual sample history and the approaches used for host identification, thereby assuming any information on the host species indicated in the available databases of sample repositories as correct or accurate. 

Molecular methods for species identification may still have critical limitations mainly due to poor sequence representation on GenBank (specifically COI databases or any other existing biodiversity databases), or any other existing biodiversity databases, of the extant mammal diversity in countries or regions with megadiverse faunas. Thus, multidisciplinary work towards the better characterization of the extant fauna is highly recommended. This should be achieved through the implementing of standard species identification tools, both taxonomic and molecular, by highly trained and experienced personnel within diagnostic laboratories worldwide. Alternatively, collaborative work between academic institutions, natural history museums and diagnostic laboratories should be fostered.

Certainly, efforts in achieving the Zeroby30 goal of eliminating dog-mediated human rabies must categorically focus on elimination interventions on dog populations. Such interventions will inevitably lead to a reduced risk for viral spillover of the RABV of dog-origin (cutting any potential for further establishment) to sympatric highly susceptible wildlife hosts. However, if new cycles of dog-origin are already established in wildlife, as current evidence suggests worldwide, rabies elimination strategies strictly focused on dog populations would have minimal to no impact in stopping emergent cycles of dog-origin in wildlife. Similarly, we do expect that the control and potential elimination of canine rabies across Africa will unveil both the public health and wildlife conservation issues related to the circulation of rabies virus in wildlife, as it occurred in other continents [38,40,41]. In that case, the scientific community should be ready to offer the appropriate investigation as well as mitigation tools to the national and international stakeholders for the early control of what could become the new major zoonotic threats.

## Figures and Tables

**Table 1:** Samples investigated for both rabies diagnosis and genetic identification of the host species. We considered a genetic confirmation of the host species for genetic identity (id) higher than 99% in the COI gene, using BOLD (indicated as BOLD-ID) or in the CytB, using BLAST (indicated as BLAST-ID). When the genetic identity was lower than 99%, the species with the highest genetic id for either COI and/or CytB, as found using BLAST, is mentioned.

Declared Host	Number of Samples	RABV Positive (Number)	Misidentification (Number)	Confirmation Family Classification	Confirmed Species
**Canids**					
canine	10	7		10	Dog (*Canis lupus*—BOLD-ID 100%)
dog	185	79	16		*n* = 1 Common patas monkey *(Erythrocebus patas*—BOLD-ID 99.5%) *n* = 5 Cats (*Felis catus*—BOLD-ID 100%) *n* = 1 Donkey (*Equus asinus*—BLAST-ID CytB 99.8%) *n* = 1 African golden wolf (*Canis anthus*—BOLD-ID 100%) *n* = 1 Goat (*Capra hircus*—BOLD-ID 99.9%) *n* = 7 Cows (*Bos taurus*—BOLD-ID 100%)
jackal	3	3		3	*Canis* sp.—BLAST with Side striped jackal (*C. adustus* BOLD-ID 98.6%, BLAST-ID 97.42%)
fox	1	1	1		*Canis* sp.—BLAST with Side striped jackal (*C. adustus*—BOLD-ID 98.6%)
**Felids**					
feline	3	2		2	Cat (*Felis catus*—BOLD-ID 99.8–100%)
cat	2	2			
wildcat	1	1	1	1	Common genet (*Genetta genetta*—BOLD-ID 100%)
lion	1	0			
**Livestock**					
bovine	14	11		14	Cow (*Bos taurus*—BOLD-ID 100%)
cow	8	8	1		*n* = 1 Dog (*Canis lupus*—BOLD-ID 100%)
caprine	8	7		8	Goat (*Capra hircus*—BOLD-ID 99.3%)
goat	4	4	3		*n* = 3 Cows (*Bos taurus*—BOLD-ID 100%)
donkey	1	1	1		*n* = 1 Dog (*Canis lupus*—BOLD-ID 100%)
**Others**					
honey badger	1	1	1		unidenfied Mustelidae—BLAST with honey badger (*Mellivora capensis*—id COI—CytB 82%) and clawless otter (*Aonyx capensis*—id COI 78.6%, CytB 84.7%)
**Total**	**242**	**127**	**24**	**38**	
**Percentage**		**52.5**	**9.9**	**15.7**	

## Data Availability

N/A.
